# Intermittent preventive treatment of malaria with sulphadoxine-pyrimethamine during pregnancy in Burkina Faso: effect of adding a third dose to the standard two-dose regimen on low birth weight, anaemia and pregnancy outcomes

**DOI:** 10.1186/1475-2875-9-324

**Published:** 2010-11-12

**Authors:** Innocent Valea, Halidou Tinto, Maxime K Drabo, Lieven Huybregts, Marie-Claire Henry, Dominique Roberfroid, Robert T Guiguemde, Patrick Kolsteren, Umberto D'Alessandro

**Affiliations:** 1 Laboratory of Parasitology and Entomology, Centre Muraz, Bobo-Dioulasso, Burkina Faso; 2 Institut de Recherche en Sciences de la santé, Direction Régionale de l'Ouest, Bobo-Dioulasso, Burkina Faso; 3 Department of Food Safety and Food Quality, Ghent University, Ghent, Belgium; 4 Unit of Nutrition and Child Health, Department of Public Health, Prince Leopold Institute of Tropical Medicine, Antwerp, Belgium; 5 Unit of Epidemiology, Department of Parasitology, Prince Leopold Institute of Tropical Medicine, Antwerp, Belgium

## Abstract

**Background:**

Intermittent preventive treatment with sulphadoxine-pyrimethamine (IPTp-SP) is being implemented in most malaria endemic countries as a standard two-doses regimen as it reduces the risk of low birth weight (LBW) and the prevalence of maternal anaemia. Nevertheless, where the risk of infection close to delivery is high because of intense transmission, a third IPTp-SP dose may further reduce the negative effects of malaria on pregnancy outcome.

**Methods:**

Pregnant women in the 2^nd ^or 3^rd ^trimester were randomized to receive either 2 (SP2) or 3 doses (SP3) of SP. Trained field workers paid home visits to the women for drug administration according to a predefined drug delivery schedule. Women were encouraged to attend their scheduled ANC visits and to deliver at the health facilities where the new-born was weighed. The prevalence of LBW (<2500 g), severe anaemia (Hb < 8 g/dL) and premature birth was analysed using intention-to-treat (ITT) and per-protocol (PP) analysis.

**Results:**

Data from 1274 singleton pregnancies were analysed (641 in the SP3 and 633 in the SP2 group). The uptake of the intervention appeared to be low. Though the prevalence of LBW in both intervention groups was similar (adjusted Incident Rate Ratio, AIRR = 0.92, 95%CI: 0.69-1.24) in the ITT analysis, the risk of severe anaemia was significantly lower in the SP3 group compared to the SP2 group (AIRR = 0.38, 95%CI: 0.16 - 0.90). The PP analysis showed a trend of reduced risk of LBW, severe anaemia and premature delivery in the SP3 group, albeit the difference between two and three IPTp-SP did not reach statistical significance.

**Conclusion:**

The risk of LBW and severe anaemia tended to be lower in the SP3 group, though this was not statistically significant, probably due to the low uptake of the intervention which reduced the power of the study. Further studies are needed for establishing whether a third SP dose has a real benefit in preventing the negative effects of malaria in pregnancy in settings where transmission is markedly seasonal.

## Background

In Africa, in malaria endemic areas, about 30 million pregnancies occur each year [1]. Though malaria infection during pregnancy is often asymptomatic where transmission is moderate to high, it increases the risk of maternal anaemia and low-birth-weight (LBW) (birth weight < 2500 g) [2-5], the greatest risk factor for neonatal mortality and an important contributor to infant mortality [6]. To prevent such adverse events, weekly chemoprophylaxis with chloroquine (CQ) has been used until recently. However, poor adherence to a weekly schedule and, more importantly, the widespread resistance to CQ [7-9], prompted its replacement with the intermittent preventive treatment with sulphadoxine-pyrimethamine (IPTp-SP) that can reduce the risk of maternal anaemia, placental malaria and LBW [10-12]. SP has a good safety profile in pregnancy and the single-dose regimen allows the direct observation of the drug intake at the antenatal clinics (ANC). Therefore, in areas of stable malaria transmission, provision to all pregnant women of at least two doses of SP after quickening has been recommended [13]. IPTp-SP is now being implemented in most African endemic countries at a standard two-dose regimen. In settings where HIV prevalence is greater than 10%, a three-dose regimen is recommended [13]. The latter may be beneficial also where malaria transmission is markedly seasonal as women receiving the second IPTp-SP dose more than one month before delivery can still be re-infected [14]. Therefore, a third dose given as late in pregnancy as acceptable, probably at 34-35 weeks gestation would prevent placental re-infection and possibly reduce the risk of LBW [12]. Though several studies investigated the effect of monthly IPTp-SP [15,16], there is little evidence that a third IPTp-SP dose offer any additional benefit compared to the standard two-dose regimen [13], particularly in a context of high SP efficacy [17,18]. Therefore, the two- and three-IPTp-SP doses regimens were evaluated and compared in two peripheral health centres in Burkina Faso and results are reported below.

## Methods

### Study settings

The study was conducted between March 2006 and July 2008 in two peripheral health centres (Koho and Karaba) located in Houndé health district. Houndé is situated at about 100 km from Bobo-Dioulasso, in west Burkina Faso. Malaria is holo-endemic with marked seasonal (June - December) transmission [19]. The district hospital and the 28 peripheral health facilities located in its catchment area cover a population of approximately 247,500 people [20]. In 2007, it was estimated that about 12,500 pregnant women were at risk of malaria. Malaria represented 38% of all consultations and 52% of hospitalizations in the health district. Attendance rates to the 2^nd ^ANC visit was estimated at 69.6%. Only 25.2% pregnant women attended the ANC before the second trimester of pregnancy. The prevalence of LBW among deliveries in health facilities was estimated at 16.0%, one of the highest figures in Burkina Faso [20]. Before its implementation as IPTp, SP resistance was low in Burkina Faso, with the PCR-adjusted treatment failure at day 28 among children 6 months-15 years of age estimated at 8.2% [21].

### Study design

This study was part of a larger one investigating the impact of a fortified food supplement (FFS) versus multiple micronutrients supplement (MMS) on pregnancy outcome [22]. Pregnant women were randomized using a factorial design in permuted blocks of four to receive either: (1) two-dose SP + FFS, (2) two-dose SP + MMS, (3) three-dose SP + FSS or (4) three-dose SP + MMS. Therefore, for malaria participants were assigned to receive either the two-dose SP (SP2 group) as recommended by the National Malaria Control Program (NMCP) in Burkina Faso, or the three-dose SP (SP3 group) regimen. Randomization numbers generated by a computer programme were printed and sealed in individual opaque envelopes that were opened only when the study physician identified an eligible subject. Drug administrations were scheduled by a field pharmacist according to the gestational age at randomization and given at home by the field workers. Nutritional supplementation consisted of a daily administration of MMS or FFS as described elsewhere [22]. The sample size was estimated on the proportion of LBW as the primary outcome. Assuming the prevalence of LBW in the control group (SP2) at 15% and a loss to follow up around 10%, 542 participants per arm should be able to show a 40% reduction (from 15% to 9%) in LBW at 80% power and 5% significance.

### Study participants

The study participants were recruited through a community-based network of 30 field workers, as described by Huybregts *et al *[22]. Briefly, during a census, all women of childbearing age in the study area were identified and registered. Monthly visits by trained field workers were carried out over the whole study area to screen for pregnant cases. When pregnancy was suspected, participants were referred to one of the two health facilities for a formal pregnancy test. Written informed consent was obtained from those agreeing to participate to the study after explaining the study purpose and procedures. Women with known hypersensitivity to SP or not planning to stay in the study area for the following two years were excluded. The study protocol was approved by the Ethical Committees of the Centre Muraz, Bobo-Dioulasso, Burkina Faso, and the Institute of Tropical Medicine, Antwerp, Belgium. The study was registered at http://ClinicalTrial.gov registry (identifier: NCT00909974).

### Clinical procedures

Women were enrolled at the first ANC visit. Demographic data and medical and pregnancy history were recorded. Clinical examination was performed and vital signs, weight, height and arm circumference were recorded. These measures were repeated at each ANC visit. Gestational age was assessed by an obstetrician by performing a trans-abdominal ultrasound foetal biometry. The field workers visited the women at home for the drug administration according to the predefined drug delivery schedule. All women received the first IPTp-SP dose at the beginning of the second trimester of pregnancy, after quickening, and the second one at the beginning of the third trimester as recommended by the NMCP. In addition, women in the SP3 group received a third IPTp-SP dose one month after the second dose if this one occurred before 34 weeks of gestation. If not, the administration of the third dose was cancelled. Women identified during the first trimester of pregnancy did not receive any anti-malarial treatment before the second trimester of pregnancy unless they had clinical malaria defined by a positive slide at any parasite density together with fever. If so, they were referred to the health facilities to receive an appropriate care. Women were paid home visits daily by the field workers who collected information about any complaint after their previous visit and recorded body temperature. In case of fever (body temperature ≥37.5°C) or history of fever since the last visit, a blood sample for parasitaemia (thick and thin blood film) and later genotyping (on filter paper) was collected. Women with malaria infection were treated with a full course of quinine (24 mg/kg/day for 7 days). Pregnant women were encouraged to attend their scheduled ANC visits and to deliver at the health facilities where the new-born was weighed and measured twice by two different members of the health staff.

### Laboratory procedures

Duplicate thick and thin blood smears were collected at different time points, i.e. at the first ANC visit, before SP administration, whenever a malaria infection was suspected and at delivery. Slides were stained with Giemsa 10% (pH 7.2) and parasites were counted against 200 WBC. Ten percent of the slides were randomly selected and sent to the Centre Muraz for quality control. Maternal haemoglobin (Hb) was measured by Hemocue (HemoCue Ltd, UK) at the first and the third ANC visits.

### Main variables and definitions

Primary outcome was LBW prevalence. Secondary outcomes were the occurrence of anaemia, miscarriage, prematurity, stillbirth and neonatal mortality. LBW was defined as birth weight < 2500 g. Anaemia and severe anaemia were defined as Hb < 11 g/dL and Hb < 8 g/dL, respectively. Miscarriage was defined as delivery before 28 weeks of gestation; prematurity as ≤37 weeks of gestation, according to the gestational age at enrolment as given by the ultrasound biometry; stillbirth as delivery of a dead baby after 28 weeks of gestation; neonatal death as death within the first 28 days of life. Adolescents were defined as women ≤ 19 years old. Malaria infection was defined as detection by microscopy of *Plasmodium falciparum *asexual stages, any density. Explanatory variables and possible confounders included the study arm, the number of SP doses received (1, 2 or 3), parity (3 groups: primigravidae, gravidae 2-3, gravidae ≥4), body mass index at enrolment, malaria infection, malaria transmission season at delivery (low transmission season from January to May and high transmission season from June to December) and the nutritional supplement group women belonged to.

### Statistical analysis

Data were double entered in a Microsoft Access^® ^database. Validation and analysis were performed using Stata/IC^® ^version 10.0 software. Only singleton pregnancies were included in the analysis. Intention-to-treat (ITT) and a per-protocol (PP) analysis were performed. Since a substantial proportion of women in the SP3 group did not received the third dose of SP, an additional individual efficacy (IE) analysis was carried out. The ITT analysis included all randomized patients for whom the outcome variables were available and the effect of the intervention was determined by comparing the SP2 group to the SP3 group, regardless of the actual number of IPTp-SP doses received by the women. The PP analysis included women randomized in the SP3 group who actually received three doses and women randomized in the SP2 group who received two doses of SP and for whom the outcomes data were available. The IE analysis divided women in groups according to the number of IPTp-SP doses actually received (one, two or three), regardless of the randomization group they belonged to.

Comparison of mean values of continuous variables was done by analysis of variance, while for categorical variables a Poisson regression model with robust standard error estimates was used to evaluate the relationship between explanatory variables and outcomes. Parity, maternal age, corrected weight status at inclusion (underweight: body mass index <18.5 kg/m^2^; or normal weight) malaria infection and malaria transmission season at delivery were included in the model as possible confounders. A *p*-value ≤ 0.05 was considered as statistically significant.

## Results

**Figure 1:**
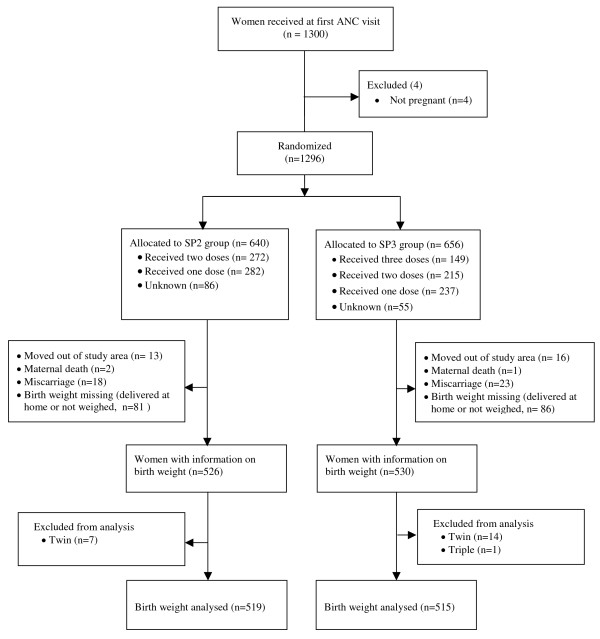
Study flow diagram

A total of 1,296 pregnant women were randomized: 656 in the SP3 and 640 in the SP2 group. However, in the SP3 group only 149 (23%) women received the third SP dose, while 261 (41%) women received the second SP dose in the SP2 group. Twenty-four women moved out of the study area and no delivery information could be obtained. For 167 women, birth weight was not available because they delivered at home and could not be referred to the health facility or the health workers failed to weigh the new-born when delivery occurred at a health facility. Twenty two pregnancies were non-singleton. Overall, the analysis of birth weight was carried out in 1,034 new-borns (Figure 1). The baseline characteristics were comparable between the two groups (Table 1). About 1/4 of the women (326/1296) were adolescents.

**Table 1:** Characteristic of study participants at inclusion by randomization group

Characteristics	SP3 group (n = 649)	SP2 group (n = 625)
Study location		
Karaba village (n, %)	152 (23.42)	173(27.68)
Koho village (n, %)	497 (76.58)	452(72.32)
Education		
none (n, %)	576 (90.85)	528(86.70)
primary (n, %)	45 (7.10)	66 (10.84)
secondary and higher (n, %)	13 (2.05)	15 (2.46)
Ethnicity		
bwaba (n, %)	160 (24.65)	165 (26.40)
mossi (n, %)	423 (65.18)	400 (64.00)
peuhl (n, %)	37 (5.70)	41 (6.56)
other (n, %)	29 (4.47)	19 (3.04)
Occupation		
farmer/housewife (n, %)	625(96.30)	609 (97.44)
student (n, %)	2 (0.31)	6 (0.96)
other (n, %)	4 (0.62)	5 (0.80)
missing (n, %)	18 (2.77)	5 (0.80)
Parity		
primigravidae (n, %)	130 (20.03)	135 (21.60)
secundigravidae (n, %)	143 (22.03)	119 (19.04)
multigravidae (n, %)	376 (57.94)	371 (59.36)
Gestation at enrolment		
first trimester (n, %)	263 (40.52)	235 (37.60)
second trimester (n, %)	345 (53.16)	343 (54.88)
third trimester (n, %)	41 (6.32)	47 (7.52)
Age		
mean (years) (95% CI)	24.26 (23.80-24.73)	24.66 (24.15-25.17)
		
Weight		
mean (kg) (95% CI]	55.40 (54.86-55.94)	55.36 (54.82-55.92)
		
Height		
mean (cm) (95% CI]	162.77 (162.32-163.25)	162.50 (162.04-162.96)
		
BMI at enrolment		
mean (95% CI]	20.88 (20.72-21.04)	20.95 (20.77-21.13)
		
Prevalence of peripheral infection	114 (17.57%)	123 (19.68%)

The prevalence of LBW was 13.44% (139/1034) and was strongly related with parity. According to ITT analysis, no difference in LBW was found between the two study groups, and this did not change after stratifying by parity (Table 2). Women of gravidity 2-3 (adjusted Incident Rate Ratio, AIRR = 0.49, 95%CI: 0.32 - 0.75), gravidity ≥ 4 (AIRR = 0.26, 95%CI: 0.15-0.47) and BMI ≥18 kg/m^2 ^(AIRR = 0.65, 95%CI: 0.44-0.96) had significantly lower risk of LBW while delivery in the high transmission season was associated with an increased risk of LBW (AIRR = 1.39, 95%CI: 1.02-1.90).

**Table 2:** Intention to treat analysis of outcomes for women randomized in the trial using Poisson regression analysis with robust error estimation (crude and adjusted Incident rate ratio).

	Prevalence (Proportion) of women with characteristics		Crude IRR (95% CI)	Adjusted IRR* (95% CI)
		
Characteristics	SP2 group	SP3 group		
**Overall**				
LBW	14.1 (73/519)	12.8 (66/515)	0.91 (0.67 - 1.24)	0.92 (0.69 - 1.24)
Anaemia	44.8 (280/625)	44.5 (289/649)	0.99 (0.87 - 1.12)	0.99 (0.88 - 1.12)
Severe anaemia	2.8 (18/625)	1.1 (7/649)	0.37 (0.16 - 0.89)**^a^**	0.38 (0.16 - 0.90)**^b^**
Premature delivery (%)	18.2 (111/610)	18.3 (116/632)	1.00 (0.79 - 1.27)	0.97 (0.77 - 1.24)
Spontaneous abortion (%)	2.8 (17/610)	3.8 (24/632)	1.36 (0.74 - 2.51)	1.36 (0.74 - 2.53)
Stillbirth	1.6 (10/610)	2.7 (17/635)	1.63 (0.75 - 3.54)	1.64 (0.74 - 3.61)
Neonatal death	2.1 (13/625)	2.8 (18/649)	1.33 (0.83 - 3.20)	1.32 (0.81 - 3.23)

**Gravidae 1**				
LBW	30.6 (34/111)	33.3(33/99)	1.08 (0.73 - 1.61)	1.05 (0.71 - 1.56)
Anaemia	47.4(64/135)	50.0 (65/130)	1.05 (0.82 - 1.35)	1.05 (0.82 - 1.34)
Severe anaemia	5.2 (7/135)	1.5 (2/130)	0.29 (0.06 - 1.40)	0.29 (0.05 - 1.46)
Premature delivery (%)	29.4 (38/129)	25.8 (33/128)	0.87 (0.58 - 1.30)	0.81 (0.55 - 1.18)
Spontaneous abortion (%)	4.7 (6/129)	8.6 (11/128)	1.84 (0.70 - 4.85)	1.51 (0.59 - 3.86)
Stillbirth	3.1 (4/129)	3.9 (5/128)	1.26 (0.34 - 4.59)	1.23 (0.30 - 4.89)
Neonatal death	5.2 (7/135)	5.4 (7/130)	1.04 (0.37 - 2.88)	0.95 (0.32 - 2.82)

**Gravidae 2-3**				
LBW	13.4 (24/179)	11.3 (21/186)	0.84 (0.48 - 1.45)	0.86 (0.50 - 1.46)
Anaemia	45.1 (97/215)	39.0 (92/236)	0.86 (0.69 - 1.07)	0.88 (0.71 - 1.09)
Severe anaemia	3.3 (7/215)	1.7 (4/236)	0.52 (0.15 - 1.75)	0.54 (0.16 - 1.84)
Premature delivery (%)	17.6 (37/210)	20.9 (48/230)	1.18 (0.80 - 1.74)	1.10 (0.75 -1.60)
Spontaneous abortion (%)	1.4 (3/209)	2.2 (5/230)	1.50 (0.36 - 6.27)	1.47 (0.38 - 5.62)
Stillbirth	0.5 (1/209)	2.6 (6/232)	5.40 (0.65 - 44.63)	5.85 (0.66 - 51.31)
Neonatal death	0.5 (1/215)	2.1 (5/236)	4.49 (1.04 - 34.32)	8.8 (1.08 - 51.49)

**Gravidae ≥4**				
LBW	6.6 (15/229)	5.2 (12/230)	0.79 (0.38 - 1.66)	0.78 (0.37 - 1.64)
Anaemia	43.3 (119/275)	46.6 (132/283)	1.07 (0.89 - 1.29)	1.06 (0.87 - 1.28)
Severe anaemia	1.5 (4/275)	0.4 (1/283)	0.24 (0.03 - 2.16)	0.24 (0.03 - 2.16)
Premature delivery (%)	13.3 (36/271)	12.8 (35/274)	0.96 (0.37 - 1.27)	0.95 (0.60 - 1.47)
Spontaneous abortion (%)	2.9 (8/272)	2.9 (8/274)	0.99 (0.37 - 2.60)	0.99 (0.37 - 2.60)
Stillbirth	1.8 (5/272)	2.2 (6/275)	1.18 ( 0.36- 3.84)	1.11 (0.34 - 3.63)
Neonatal death	1.8 (5/275)	2.1 (6/283)	1.16 (0.35 - 3.78)	1.11 (0.34 - 3.62)

*Adjusted for age category, BMI, malaria infection, malaria season at delivery, supplementation and parity category (the later for the overall analysis only), **^a ^**p= 0.026, **^b ^**p = 0.030

The prevalence of anaemia and severe anaemia in the third trimester of pregnancy was 44.66% (569/1274) and 1.98% (25/1274), respectively. Severe anaemia occurred significantly less in the SP3 than in the SP2 group (AIRR 0.38, 95%CI: 0.16 - 0.90). No difference in anaemia between SP3 and SP2 was found. About 18% (227/1242) women delivered prematurely; there were 41 spontaneous abortions (3.3%), 27 stillbirths (2.17%), and 31 neonatal deaths (2.43%). For these outcomes, no difference between the study groups was found (Table 2).

According to the PP analysis, the proportion of LBW (2-dose SP: 11.9%, 3-dose SP: 9.6%), severe anaemia (2-dose SP: 2.3%, 3-dose SP: 1.7%) and premature delivery (2-dose SP: 11.3, 3-dose SP: 9.5%) tended to decrease with the number of SP doses received, albeit it did not reach statistical significance (Table 3), and this occurred also for most outcomes after stratifying by gravidity.

**Table 3:** Per Protocol analysis of pregnancy outcomes for women randomized in the trial using Poisson regression analysis with robust error estimation (crude and adjusted incident rate ratios)

	Prevalence (Proportion) of women with characteristics		Crude IRR (95% CI)	Adjusted IRR* (95% CI)
		
Characteristics	2-dose SP	3-dose SP		
**Overall**				
LBW	11.9 (28/235)	9.6 (13/136)	0.80 (0.42 - 1.49)	0.84 (0.45 - 1.54)
Anaemia	46.1 (119/258)	50.7 (75/148)	1.09 (0.89 - 1.35)	1.12 (0.91 - 1.38)
Severe anaemia	2.3 (6/258)	1.4 (2/148)	0.58 (0.12 - 2.84)	0.63 (0.12 - 3.46)
Premature delivery (%)	11.3 (29/257)	9.5 (14/148)	0.83 (0.46 - 1.53)	0.77 (0.43 - 1.35)
Spontaneous abortion (%)	1.2 (3/257)	2.0 (3/148)	1.73 (0.35 - 8.51)	1.56 (0.34 - 7.10)
Stillbirth	1.2 (3/257)	2.7 (4/148)	2.31 (0.5 - 10.22)	2.32 (0.47 - 11.36)
Neonatal death	1.3 (2/136)	1.5 (2/136)	1.15 (0.19 - 6.82)	1.29 (0.21 - 7.99)

**Gravidae 1**				
LBW	19.2 (9/47)	13.6 (3/22)	0.71 (0.21 - 2.39)	0.78 (0.22 - 2.72)
Anaemia	63.5 (33/52)	58.3 (14/24)	0.92 (0.62 - 1.36)	1.00 (0.68 - 1.49)
Severe anaemia	3.9 (2/52)	0	-	-
Premature delivery (%)	17.3 (9/52)	12.5 (3/24)	0.72 (0.21 - 2.45)	0.56 (0.15 - 2.09)
Spontaneous abortion (%)	3.9 (2/52)	4.2 (1/24)	1.08 (0.10 - 11.52)	0.31 (0.08 - 1.25)
Stillbirth	1.9 (1/52)	0	-	-
Neonatal death	4.3 (2/47)	0	-	-

**Gravidae 2-3**				
LBW	15.4 (12/78)	11.5 (6/52)	0.75 (0.29 -1.87)	0.81 (0.32 - 2.01)
Anaemia	41.7 (35/84)	43.9 (25/57)	1.05 (0.71 - 1.55)	1.10 (0.73 - 1.66)
Severe anaemia	2.4 (2/84)	1.8 (1/57)	0.89 (0.06 - 8.00)	0.92 (0.10 - 8.77)
Premature delivery (%)	15.5 (13/84)	12.3 (7/57)	0.79 (0.34 - 1.87)	0.77 (0.35 - 1.68)
Spontaneous abortion (%)	0	1.8 (1/57)	-	-
Stillbirth	0	5.3 (3/57)	-	-
Neonatal death	0	0.8 (1/130)	-	-

**Gravidae ≥4**				
LBW	6.4 (7/110)	6.5 (4/62)	1.01 (0.31 - 3.34)	1.09 (0.32 - 3.68)
Anaemia	41.8 (51/122)	53.7 (36/67)	1.28 (0.95 - 1.75)	1.26 (0.92 - 1.71)
Severe anaemia	1.6 (2/122)	1.5 (1/67)	0.91 (0.08 - 9.91)	-
Premature delivery (%)	5.8 (7/121)	5.9 (4/67)	0.72 (0.21 - 2.45)	-
Spontaneous abortion (%)	0.8 (1/121)	1.5 (1/67)	-	-
Stillbirth	1.6 (2/121)	1.5 (1/67)	0.90 (0.08 - 9.83)	0.77 (0.04 - 15.87)
Neonatal death	0.9 (1/110)	1.6 (1/62)	1.77 (0.11 - 2.80)	-

*Adjusted for age category, BMI, malaria infection, malaria season at delivery, supplementation and parity category (the later in the overall analysis only)

For the IE analysis, three groups of women were defined: those having received one dose (515), two doses (472), and three doses (149). Women were excluded when the number of SP doses received was unknown (138/1296, 10.65%).

In primigravidae, the risk of LBW was significantly different between those having received one (41.4%, 36/87), two (25%, 21/84) and three IPTp-SP (13.6%, 3/22) doses (*p = 0.012*), though the difference between two and three IPTp-SP doses did not reach statistical significance (*p = 0.47*). Similarly, in this subgroup, the mean birth weight was significantly different between primigravidae having received one (2570 g), two (2703 g) or three IPTp-SP (2821 g) (*p = 0.04*) but not between those having received two and three doses (*p = 0.87*). The risk of severe anaemia in the third trimester (one 3.6%, 4/110, two 2.9%, 3/111, three 0.0%, 0/24) and of premature delivery (one 33.0%, 36/106; two 20.0%, 20/100; three 12.0%, 3/24) was also significantly different according to the number of IPTp-SP doses taken but not between those having received two and three doses (data not shown). In all other gravidae, no difference according to the number of IPTp-SP could be found.

## Discussion

This study had the objective of establishing the benefits of adding a third SP dose to the standard two-dose IPTp-SP regimen on pregnancy outcome. However, a significant proportion of women randomized to the SP3 group did not receive the third SP dose and this reduced substantially the power of the study. Unfortunately, such problem was identified only at the time of the statistical analysis, when no corrective action could be taken. The trial comparing SP2 against SP3 was carried out within a larger study looking at nutritional supplementation during pregnancy, in which women had to be visited daily by field workers. This was thought to be a major advantage as women should have been carefully monitored and the timing of SP administration planned well in advance. Conversely, such careful follow up did not translate in high coverage of the intervention, rather in an extremely low uptake of the third SP dose. The reasons for such results are unclear but definitely cannot be attributed to the low performance of the health system as the intervention was delivered by field workers supervised by the local research team. It should be noted that randomization was not stratified by gestational age and this may have resulted in the inclusion of late pregnancies in which the third dose could not be administered before delivery. Nevertheless, most pregnant women were identified relatively early, mainly during the second trimester, and should have been able to receive the third SP dose. The more likely explanation is that this was an ancillary study, carried out within a larger nutrition project, and that the local research team probably either misunderstood the drug administration schedule, failing to provide SP at the required time, or was 'too busy' in managing the large amount of data produced by the daily visits. The low coverage is clearly a major weakness of this study though it should be considered that most of the SP third doses were administered to women randomized to the SP3 group. Therefore, a major selection bias is unlikely; rather the study has a lower power than expected and is unable to provide robust conclusions about the benefit of the third SP dose. This is obvious when considering the results obtained by the ITT and PP analysis. In the former no difference in the occurrence of LBW and anaemia was found, an expected result when considering the substantial number of pregnant women in SP3 group having received just 1 or 2 SP doses, diluting any possible difference. In the PP analysis, the risk for LBW, severe anaemia and premature delivery tended to be lower in the SP3 as compared to the SP2 group though, due to the small sample size and hence the low power, such difference does not reach statistical significance, a result illustrated by the wide AIRRs' 95% confidence intervals. Therefore, the study results are inconclusive in regards of the potential benefits of a third IPTp-SP dose on reducing the risk of LBW.

Nevertheless, the decreasing trend of LBW, severe anaemia and premature delivery risk observed in the PP analysis may be an indication of the possible additional effect of the 3^rd ^dose of SP. The significantly lower risk of severe anaemia in the SP3 group could be considered as an indication of the beneficial effect of the third SP dose though this was not confirmed by the PP analysis, most likely because of the small number of women included in this analysis. A reduction in the risk of severe anaemia is plausible as, though of complex aetiology, known causes including worm infections, nutrient deficiencies and chronic inflammation [23], maternal anaemia has been also associated to placental malaria [23] so that a third SP dose, by reducing such a risk, could have also improved the haematological status. The nutritional supplements received by the women included in the study should have further reduced the risk of maternal anaemia by preventing folic acid and iron deficiency, the latter frequently observed in sub-Saharan African women [24]. Indeed the daily dose included at least 400 μg of folic acid and 30 mg of iron as part of the FFS or MMS.

In the IE analysis, the effect of two or three doses of IPTp-SP was more evident in primigravidae. In areas of intense transmission, their higher vulnerability to malaria infection and its negative consequences are well known [5]. In these settings, any intervention against malaria should have a more profound effect in this group than in secundi- and multigravidae. Indeed, in a study on the effectiveness of IPTp-SP carried out in Burkina Faso, in a similar setting, the impact of two IPTp-SP doses as compared to one or none was more marked in primigravidae than in secundigravidae [17]. In addition, according to a Cochrane review, anti-malarial drugs reduce significantly severe antenatal anaemia and LBW, and increase birth weight in women in their first and second pregnancy [25].

In conclusion, no difference on LBW risk was found by adding a third dose of SP to the standard 2-dose regimen, though a trend of reduced risk was observed. Such finding does not exclude a beneficial effect of a third IPTp-SP dose, rather additional and more carefully carried out studies are needed to establish the potential benefits and the feasibility of this strategy in areas of intense malaria transmission.

## Competing interests

The authors declare that they have no competing interests.

## Authors' contributions

IV participated in study coordination and data cleaning, performed the data analysis and drafted the manuscript. HT participated in the design, study coordination and corrected the manuscript. MKD corrected the manuscript. LH participated in data cleaning and corrected the manuscript. DR, MCH, RTG and KP participated in the design of the study. UDA participated in the design and helped to analyse the data and draft the manuscript. All authors read and approved the final manuscript.
